# Utility of Virtual New-Patient Breast Surgery Clinics in Women Under 30 Years Old

**DOI:** 10.7759/cureus.93779

**Published:** 2025-10-03

**Authors:** Jacob N Morton, Hudhaifah Shaker, Nabila Nasir

**Affiliations:** 1 Department of Breast Surgery, North Manchester General Hospital, Manchester, GBR

**Keywords:** benign breast condition, breast cancer detection, oncoplastic breast surgery, one-stop clinic, telemedicine, virtual consultations

## Abstract

Background: Virtual consultations (VCs) have been utilized across various clinical specialties and have the potential to reduce outpatient clinic costs and improve patient satisfaction. In breast surgery, we aimed to assess the utility and feasibility of VC in new-patient clinics for women under 30, who are considered a low-risk subgroup.

Methods: Between December 2020 and April 2021, data were consecutively collected from 118 women aged under 30 who were referred from primary care to the breast clinic. Participants were offered VCs, followed by ultrasound (USS) if clinically indicated and/or face-to-face review. Clinicopathological data were collected on referrals, imaging, and follow-up, with descriptive statistics used for analysis.

Results: An outpatient USS was performed by a sonographer trained in palpation for 80.3% (n = 94) of patients after a review in the VC clinic. In comparison, 15.4% (n = 18) of patients were reassured and discharged directly after the VC consultation. The USS was normal in 64.9% (n = 61) of patients, with the remainder showing benign pathology. Six biopsies were performed, all of which were benign. Only 14.4% (n = 17) of patients required a face-to-face appointment with a breast surgeon, and 64.1% (n = 75) of patients were discharged after VC + USS alone. Routine follow-up of benign disease was organized for 6.0% (n = 7) of patients, with one patient booked for elective surgery for benign disease. No adverse events were reported.

Conclusion: Utilizing VC, the majority of new referrals did not require face-to-face appointments, and the diagnosis rate is low in this younger age subgroup. VCs have the potential to reduce the burden on new-patient clinics and reduce costs while improving patient convenience. Larger studies with longer follow-up are needed to assess the long-term safety of this approach.

## Introduction

Virtual consultations (VCs), also known as online or telemedicine services, are increasingly utilized in healthcare settings [1]. There are multiple benefits to running clinics virtually. Virtual clinics significantly reduce the environmental impact and financial burden to patients traveling to appointments [2,3]. They are generally less expensive for service providers than face-to-face clinics [3]. Studies assessing patient experience and satisfaction at virtual follow-up cancer clinics have found virtual clinics to be equivalent [4-6] or, in some cases, preferable [7] to face-to-face counterparts.

In response to COVID-19 restrictions, the Breast Unit at North Manchester General Hospital implemented virtual follow-up clinics [8]. Following local success and early supportive data for telemedicine in preoperative and postoperative care [9], the unit subsequently introduced virtual new patient clinics to assess subgroups considered low risk for malignant disease.

Epidemiological data from 2016-2018 report the incidence of breast carcinoma in situ in the UK in the female population under 30 per year to be approximately one in 10,000 [10]. Most patients in this subgroup at North Manchester General Hospital have normal scans or benign disease, and nearly all are discharged after an ultrasound (USS) scan alone. One-stop, triple-assessment clinics remain the gold standard [11]. However, for women under 30 who do not require mammograms, a separate virtual clinical assessment and USS scan performed by sonographers and radiologists may be appropriate.

VCs have been safely used in two-week wait pathways, including skin cancer [12] and colorectal cancer [2] pathways. However, these pathways have different screening and examination requirements than breast services. In the postpandemic era, VC has been utilized in other medical specialties as an alternative to usual care [13-15]. Now that the pandemic has subsided, the question arises whether the benefits of VCs can be retained as part of regular breast services, particularly in low-risk subgroups.

This study examines women under 30 years of age referred for potential breast tumors. The utility and feasibility of VCs in this population are not well established, and this study aims to address this gap.

## Materials and methods

Departmental approval was obtained to establish a new patient clinic for women under 30 years of age. As per local policy, ethical approval was not required for retrospective case note reviews. This initiative followed the implementation of video follow-up clinics during the COVID-19 pandemic in early 2020.

Study design

This cross-sectional study analyzed 118 women under 30 years of age referred to a breast clinic with new breast symptoms. All patients aged 18-30 referred to the breast surgery new-patient clinic at North Manchester General Hospital between December 2020 and April 2021 were included. The participants were enrolled consecutively.

Exclusion criteria included patients previously seen or treated by the breast team within the last six months, male patients, and patients booked for VCs who had a face-to-face appointment as their initial appointment. Patients were referred to the breast clinic by primary care doctors (general practitioners, GPs) based on locally agreed-upon guidelines.

Assessment in the clinic

All patient referrals were triaged by a consultant breast surgeon before allocation to a virtual new patient clinic to confirm eligibility based on age and absence of prior contact with the breast team within the preceding six months. Patients were assessed by a consultant surgeon and offered a choice of telephone or video assessment using a smartphone. Each VC lasted 10 minutes and was conducted via hospital tablets or telephones, including a comprehensive breast history and, when possible and appropriate, a video examination.

Based on the initial consultation, assessing surgeons decided to either 1) reassure and discharge, 2) arrange a face-to-face clinical assessment by a surgeon, 3) arrange a focused USS by an experienced sonographer with experience in clinical palpation, or 4) arrange both a face-to-face clinical assessment by a surgeon and a focused USS.

All discharged patients were given routine safety-netting advice and available re-referral via the two-week-wait pathway if the GP had further concerns. For patients referred directly for USS, surgeons reviewed the reports and determined whether further follow-up was required, either in person or virtually, or if the patient could be safely discharged. Patients who underwent USS-guided biopsy or fine-needle aspiration were discussed at a multidisciplinary meeting that included surgeons, pathologists, and radiologists.

Data collection and analysis

Clinicopathological data were collected on referrals, imaging details, USS scan outcomes, biopsy results, final outcomes, and any required follow-up. All data were initially entered into Microsoft Excel (Microsoft Corporation, Redmond, WA) and subsequently coded for analysis using IBM Statistical Package for the Social Sciences Statistics version 29.0 (IBM Corp., Armonk, NY). Descriptive statistics were used for data analysis. Data collection occurred six months after consultation.

## Results

A total of 118 patients were identified during the study period, with a median age of 24 years (range 17-30). One patient was excluded due to insufficient records.

Virtual clinic referral and presentation

All patients were referred by their primary care provider (GP) to the breast unit. The most frequently reported symptom was a lump, followed by breast pain. Additional symptoms included skin changes, nipple changes, nipple discharge, and axillary symptoms (see Table 1).

**Table 1:** Presenting symptom

Symptom	Frequency (n)	Percentage (%)
Lump	82	69.5
Breast pain	61	53.1
Skin changes	11	10.2
Nipple changes	9	7.0
Nipple discharge	7	5.5
Axilla symptoms	7	5.5

Details of the VC

Virtual secondary care consultations were conducted by a consultant breast surgeon, with 53.8% (n = 63) conducted via video and 41.0% (n = 48) by telephone. In 5.1% (n = 6) of cases, the consultation mode was unclear from the documentation. Among the 62 patients who had a video consultation, 45.2% (n = 28) underwent a video examination.

Outcome of VC

**Figure 1:**
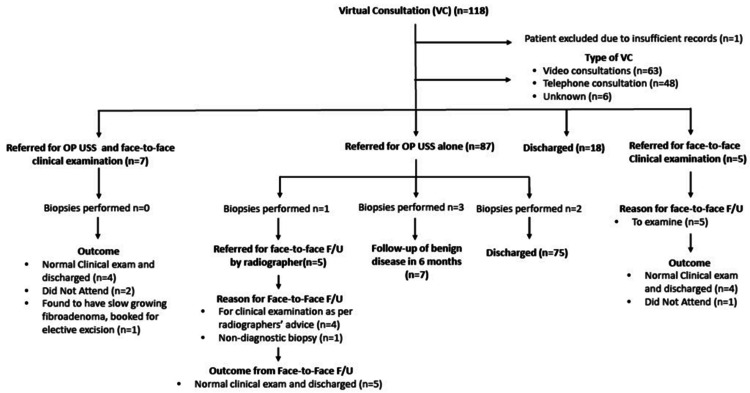
Patient journey flowchart VC: virtual consultation; OP USS: outpatient ultrasound; F/U: follow-up

The following flowchart (see Figure 1) summarizes the outcome of the VC and patient pathway.

The outcomes of VCs were similar regardless of whether patients received video or telephone consultations. The only notable difference was that 10.4% (n = 5) of the telephone consultation cohort were referred for face-to-face review alone. In contrast, none from the video consultation cohort were referred for face-to-face review alone. Among video consultations, outcomes were consistent regardless of whether a video examination was performed.

Radiology and pathology findings

Overall, 80.3% (n = 94) of patients underwent a USS scan. In 64.9% (n = 61) of cases, the USS revealed no discrete lesions or only fibrocystic changes consistent with a nonpathological examination. The most common clinical finding was fibroadenoma, identified in 17.0% (n = 16) of scans. No USS scans identified lesions suspicious for malignancy (see Table 2).

**Table 2:** USS findings USS: ultrasound

USS findings (n = 94)	Frequency (n)	Percentage (%)
No discrete lesion/fibrocystic change	61	64.9
Fibroadenoma or preexisting fibroadenoma	16	17.0
Simple cysts	3	3.2
Dermal/sebaceous cyst	4	4.3
Axilla/chest normal node	2	2.1
Other	4	4.3
DNA	3	3.2
USS was not performed for an unknown reason	1	1.1

Among the 94 patients who underwent ultrasonography, 6.7% (n = 6) required a biopsy as determined by a sonographer trained in clinical examination. All biopsies demonstrated benign disease (see Table 3).

**Table 3:** Biopsy result

Biopsy result (n = 6)	Frequency (n)	Percentage (%)
Fibroadenoma	3	50.0
Lactating adenoma	1	16.7
Indeterminate cells	1	16.7
No abnormal cells	1	16.7

Face-to-face follow-up

Of the 118 patients who attended the virtual new patient clinic on a two-week wait pathway, 14.4% (n = 17) required face-to-face assessment. Patients were referred either directly from a VC for face-to-face assessment with or without an USS scan, or following an USS scan based on the radiographer's recommendation or biopsy findings. Further details are provided in Figure 1.

Among the 17 patients referred for face-to-face follow-up, 17.6% (n = 3) did not attend, 76.5% (n = 13) had a normal clinical examination and were discharged, and one patient (5.9%) was booked for elective excision of a fibroadenoma (see Table 4).

**Table 4:** Outcome of face-to-face assessment

Outcome of face-to-face assessment (n = 17)	Frequency (n)	Percentage (%)
Normal clinical exam and discharged	13	76.5
Booked for elective excision of fibroadenoma	1	5.9
Did not attend	3	17.6

Final outcome

Of the 117 patients seen in the virtual two-week wait clinic, 15.4% (n = 18) were discharged directly back to primary care from the VC without face-to-face or radiological assessment, based on a consultant's low index of suspicion for breast disease. None of these patients presented with a new, discrete lump. An additional 64.1% (n = 75) were discharged after the VC and USS scan. A further 7.7% (n = 9) were discharged after a VC, USS scan, and face-to-face assessment. A total of 2.6% (n = 3) did not attend their face-to-face assessment. Eight patients remained under the breast service for benign disease. Of these, 3.4% (n = 7) were scheduled for follow-up in six months, and 0.9% (n = 1) were booked for elective operative management (see Table 5). None of the discharged patients re-presented within six months after consultation.

**Table 5:** Final outcome VC: virtual clinic; USS: ultrasound

Final outcome (n = 117)	Frequency (n)	Percentage (%)
Discharged directly from the VC	18	15.4
Discharged after VC + USS alone	75	64.1
Discharged after VC + USS and face-to-face assessment	9	7.7
Discharged following VC + face-to-face assessment alone	4	3.4
Booked for follow-up of benign disease in six months	7	6.0
Did not attend face-to-face follow-up	3	2.6
Booked for elective excision of fibroadenoma	1	0.9

## Discussion

One-stop triple assessment clinics are the current gold standard practice used by National Health Service (NHS) services for new patients referred with suspected breast cancer [11]. These clinics present significant logistical and financial challenges. They require staffing by surgeons or advanced nurse practitioners trained in clinical assessment, as well as radiologists or advanced sonographers, and need substantial clinical space. Previous evidence suggests one-stop clinics may be less cost-effective than dedicated breast clinics and may not significantly alleviate patient anxiety, despite being the NHS gold standard [16].

This study evaluated the feasibility of conducting a new patient symptomatic breast clinic virtually, without requiring a face-to-face examination at initial contact. The focus was on a low-risk subpopulation in whom cancer diagnosis is rare and rapid diagnosis is infrequently necessary [17,18]. The findings indicate that a substantial majority of patients completed their consultation and diagnostic workup without face-to-face appointments. This approach may offer a more convenient alternative for patients, reduce the burden on outpatient services, and enable more efficient resource allocation.

Significant challenges are associated with transitioning to VCs. It is essential to acknowledge that VCs lack the physical examination by a consultant breast surgeon. Older patient subgroups have expressed mixed views regarding the absence of a physical exam during VCs, particularly when presenting with new complaints [19]. The impact of omitting a consultant-led physical examination in the diagnostic pathway for women under 30 requires further evaluation to ensure safe implementation of virtual new patient clinics. It is notable that 15.4% of patients in this pathway were discharged without in-person review or radiological imaging. None of these patients presented with a new, discrete lump, and no adverse events were reported at six months; however, the long-term impact of this approach requires further study.

The widespread integration of VC technologies into current practice will require a substantial commitment across all levels. Early implementation stages are likely to necessitate local in-person support and troubleshooting to address technical and operational challenges [20,21]. Healthcare trusts must provide comprehensive training [22] and reskilling of staff. Strict information governance policies are essential to protect privacy and confidentiality [20]. Additionally, the NHS technical infrastructure and IT systems must be sufficient to support reliable VCs [23].

Despite these challenges, given the previously outlined benefits of VC for patients and service optimization, efforts to assess the utility of VC are worthwhile. The implementation of VCs at scale in high-volume tertiary cancer centers may be feasible [24]. Multiple large-scale studies have reported high rates of patient satisfaction with VCs, and many patients prefer this modality [25,26]. A survey at North Manchester General Hospital indicated that over 83% of patients rated the VC process as “better” or “similar” to face-to-face, and 90% reported saving between one and four hours by not attending in person.

Limitations

These findings should be interpreted with caution. The small sample size and short follow-up period limit the generalizability of the results. Although the study demonstrated the feasibility of VCs, the ability to diagnose malignancies via remote assessment remains uncertain. The diagnostic accuracy of VCs in breast cancer was not assessed, which is a critical aspect of breast health assessments. Given the low incidence of malignancy in this age group, the absence of cancer at six months is expected. As such, conclusions regarding diagnostic safety or equivalence to the gold standard triple assessment clinic cannot be drawn.

The study methods possess inherent limitations. The absence of standardized protocols for VCs and sonographer training reduces reproducibility. Additionally, patient experience data were not collected. Reliance on descriptive statistics further limits the strength of the study's conclusions.

The study did not examine potential delays in treatment or follow-up for patients who required face-to-face consultations compared to a one-stop model. Additionally, as only women under 30 years of age were included, these findings are not generalizable to older or higher risk patient subgroups.

Further research with a larger patient cohort and extended follow-up is necessary. Future studies should assess the diagnostic accuracy of virtual clinic pathways for breast cancer detection.

While VCs may offer significant benefits, they also present inherent limitations. The inability to engage in face-to-face conversations or perform physical examinations may hinder the establishment of trust and effective communication, particularly in shared decision-making for complex cases. Proposed models of VC may improve shared decision-making in this context [27]. Further research is required to evaluate the role of VC in preoperative and postoperative care, especially for patients requiring surgical intervention.

## Conclusions

VCs appear feasible for low-risk women under 30 years of age. In this cohort, many patients were managed without face-to-face consultations, and VCs may offer increased convenience and potential cost-effectiveness. This model may provide a pragmatic alternative for managing low-risk patients, particularly when combined with targeted USS assessment and clinician oversight. However, due to the small sample size, short follow-up, and reliance on descriptive statistics, conclusions regarding long-term safety and diagnostic accuracy cannot be made. Prospective studies with larger cohorts and extended follow-up are necessary before widespread adoption.

These findings highlight the need for careful selection of appropriate patient groups and robust clinical governance to ensure the safe expansion of VCs. The absence of an initial in-person examination must be balanced with clinical context, risk stratification, and timely access to imaging or in-person review when necessary. Further research with larger cohorts and extended follow-up is essential to fully understand the long-term impact on patient safety, diagnostic accuracy, and quality of care.
